# Draft Genome Sequence of Stenotrophomonas maltophilia CRB139-1, Isolated from Poultry Meat in Japan

**DOI:** 10.1128/MRA.00075-20

**Published:** 2020-03-19

**Authors:** Shiori Yamamoto, Tatsuya Nakayama, Hiroshi Asakura

**Affiliations:** a Division of Biomedical Food Research, National Institute of Health Sciences, Kawasaki-ku, Kawasaki, Kanagawa, Japan; University of Southern California

## Abstract

Stenotrophomonas maltophilia is a nosocomial pathogen that primarily causes respiratory infection in humans. This pathogen is widely distributed in the environment, including in foods. Here, we report the draft genome sequence of S. maltophilia strain CRB139-1, isolated from poultry meat in Japan. The genome size was 4,619,918 bp at 90× coverage.

## ANNOUNCEMENT

Stenotrophomonas maltophilia is a nosocomial pathogen that causes respiratory infection in humans (1, 2). This bacterium resides in animal hosts and in a number of different environments and food specimens (2–6). Most available genomic data were obtained from human or environmental specimens, and there is limited information available on the strains found in food; there are only two partial sequences (GenBank accession numbers KU978825 and MH450104) for the genomic data of strains from poultry meat. To address the lack of genomic data, we obtained *S. maltophilia* strain CRB139-1, a single colony isolated from violet-red bile glucose agar containing 2 µg/µl meropenem, from poultry meat in Hiroshima, Japan, in 2015. This study obtained the genome sequence of CRB139-1 to explore the strain’s genetic background.

**FIG 1:**
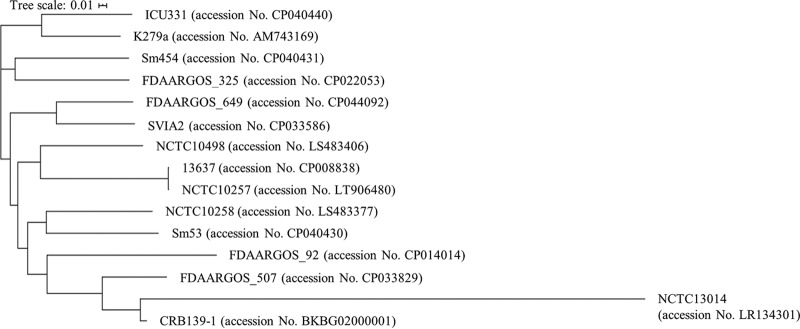
Dendrogram of the genetic phylogenetic tree obtained from the whole-genome sequences of 15 representative S. maltophilia strains, including isolate CRB139-1. The complete genome sequences of 15 S. maltophilia reference strains were obtained from DDBJ/EMBL/GenBank. The phylogenetic tree was constructed using the concatenated alignment of the single nucleotide polymorphisms (SNPs) in whole-genome sequencing reads in CSI phylogeny v.1.4 with default parameters and was displayed by Interactive Tree of Life (iTOL).

Genomic DNA was extracted from a bacterial culture grown in lysogeny broth agar (Becton, Dickinson) using the Maxwell RSC blood DNA kit (Promega), and the library was prepared using the Ion Xpress Plus fragment library kit (Life Technologies). Genome sequencing was performed using the Ion Torrent Chef/GeneStudio S5 system (Life Technologies). The reads were trimmed using Qiagen CLC Genomics Workbench v.11.0 and assembled *de novo* to the contig and scaffold levels using CGE Assembler v.1.2 and CONTIGuator v.2 (7, 8) with the reference genome sequence of strain NCTC13014, as this was shown to be the closest relative of CRB139-1 (Fig. 1). The draft genome was annotated using the DDBJ Fast Annotation and Submission Tool v.1.1.4 (9). Default parameters were used for software unless otherwise specified. Identities to other strains were determined using the NCBI BLAST tool.

Sequence data comprised 461,320,092 bp from 1,895,353 reads and were assembled into 489 contigs, with an *N*_50_ value of 17,160 bp. The genome sequence was embedded in one scaffold with 0.854% gaps and an accumulated length of 4,619,918 bp at 90× coverage. The assembly had a G+C content of 66.3% and an *N*_50_ value of 4,619,918 bp. Scaffold sequences were annotated to contain 4,215 coding sequences.

S. maltophilia is known to be intrinsically resistant to carbapenems via β-lactamase production or multidrug efflux pumps (10). Strain CRB139-1 harbored the *bla*_L2_ gene, which is associated with decreased production of β-lactamase (11); this gene was 100% identical to that of strain FDAARGOS_507. Mutations in the *bla*_L2_ gene or AmpR regulator result in overproduction of β-lactamase (12, 13). AmpR, which activates the transcription of *bla*_L2_ (14, 15), showed four amino acid substitutions compared to that of strain KH (99.3% similarity). The start codons and promoters of these genes and a putative AmpR binding region (15) were identical to those of KH, whereas the similarity with *ampR*-*bla*_L2_ intergenic sequences was 91.6%. Previous studies indicated that *ampR*-*bla*_L2_ sequence variation is involved in L2 β-lactamase expression (16–18). Thus, our data suggest that a certain mutation(s) in these regions might be associated with increased meropenem resistance.

This study provides genome sequence data of S. maltophilia isolated from poultry meat. Further comparative genomic analysis would elucidate the molecular mechanisms underlying the growth of this bacterium in poultry meat.

### Data availability.

These sequences were deposited in DDBJ/ENA/GenBank under accession number BKBG02000001 and the raw reads under accession number DRA008814, with BioProject number PRJDB8492.
